# Use of GIS and Exposure Modeling as Tools in a Study of Cancer Incidence in a Population Exposed to Airborne Dioxin

**DOI:** 10.1289/ehp.6739

**Published:** 2004-04-15

**Authors:** A. Poulstrup, H.L. Hansen

**Affiliations:** Regional Public Health Office, Vejle, Denmark

**Keywords:** air pollution, cancer, dioxin, environmental epidemiology, exposure model, GIS, health registers, migration

## Abstract

In environmental health research there is a recognized need to develop improved epidemiologic and statistical methods for rapid assessment of relationships between environment and health. Exposure assessment is identified as a major challenge needing attention. In this study an exposure simulation model was used to delimit almost exactly in space and time an urban population exposed to airborne dioxin. A geographic information system (GIS) was used as the electronic environment in which to link the exposure model with the demographic, migration, and cancer data of the exposed population. This information is available in Denmark on an individual basis. Standardized incidence ratios (SIRs) for both men and women in 10-year age bands were calculated for three different exposure areas. Migration patterns were outlined. SIRs showed no excess of cancer incidences during the time span chosen (13 years; 1986–1998) in the whole exposed area or in the medium or higher polluted areas. The exposure model appeared very useful in selection of the appropriate exposure areas. The integration of the model in a GIS together with individual data on addresses, sex, age, migration, and information from routine health statistics (Danish Cancer Registry) proved its usefulness in demarking the exposed population and identifying the cancers related to that population. Less than one-third of the study population lived at the same address after 13 years of observation, and only half were still residing in the area, indicating migration of people as a major misclassification.

A major challenge for investigators in environmental epidemiology is to correctly identify populations at risk from exposure to environmental contaminants. To date, three methods have been used to identify the populations at risk from point sources of air pollution: physical monitoring, environmental monitoring, or mathematical modeling (Williams and Ogston 2002). This article is a discussion of the utilization of a computerized air pollution model, normally used by the environmental protection authorities for assessing pollution values (immissions), and the putative offense of legally set thresholds of emission. To test the model for its appropriateness as an improved tool for assessment of exposure, an actual case was used of known dioxin air pollution in an urban area.

In the town of Kolding in the southern part of the Jutland peninsula, Denmark, three outlets of dioxin were identified. All three emitted dioxin into the air through their chimneys. The dioxin consisted mainly of 2,3,7,8-tetrachlorodibenzo-*p-*dioxin (TCDD, or dioxin). One outlet in particular, an aluminum recycling plant, was found on two occasions—early November and early December 2000—to have emitted large quantities of dioxins, up to 180 ng/m^3^/hr. All three plants had been operating for years. The main culprit was the aluminum recycling plant, which had been in continuous operation since 1970 with an almost unchanged method of production and output.

Our plan was to layer the computer-simulated exposure model in a geographic information system (GIS) and use the simulated immission concentrations to more accurately demarcate the exposed population. The information on addresses, vital statistics, migration, and cancer of the population of Denmark or any subset was available on individuals and on the delineated population in this study. This information was layered into the same GIS environment, enabling a more exact identification of the exposed population in both space and time.

All malignant cancers were used as the health indicators of the exposed population to assess eventual negative health outcomes caused by the dioxin pollution. TCDD is a major environmental carcinogen causing various types of cancers (IARC 1997).

## Materials and Methods

The air pollution simulation model used in Denmark to assess hourly immissions of airborne pollutants is a Gaussian air dispersion model based on emission data of the actual pollutant(s) and time series of meteorological data such as wind speed, wind direction, wind temperature, rain, snow, number of stacks, their heights, surrounding buildings, and surrounding terrain. The model [OML, Operationel Meteorologiske Luftkvalitets-modeller (Danish)] has been widely validated both in Europe and North America and is reliable in predicting hourly immissions of one or more airborne pollutants (DMU 2001). Only two measurements of dioxin were available as input source, and both were obtained in November and December 2000. The GIS used was the software package ArcGis version 8.12 (ESRI, Atlanta, GA, USA).

**Figure 1:**
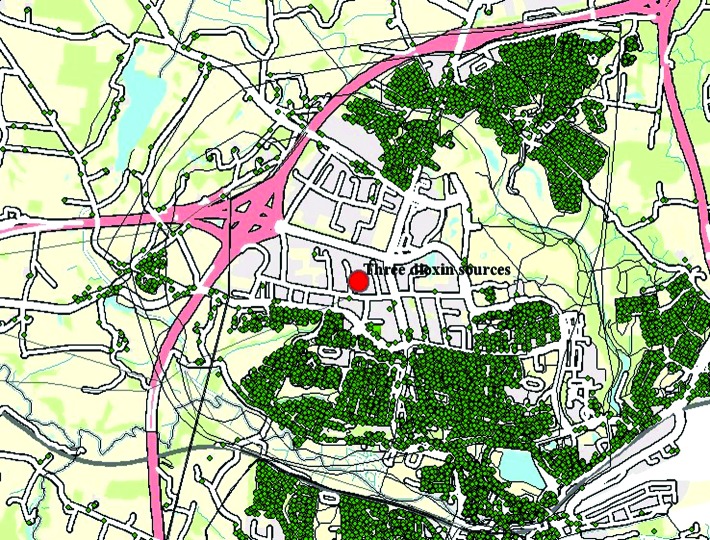
Map of Kolding Town with dioxin source in red and address points in green .

All current and past addresses in Denmark since 1999 were geocoded with Universal Transverse Mercator coordinates with a precision of a few meters and subsequently layered into the GIS. The Address Project is described elsewhere (Briggs et al. 2002). By linkage of all individuals to these addresses using the unique Central Population Register (CPR) number (10-digit number in the Civil Registration System), the GIS eventually contained all the following information in addition to the addresses of each individual: date of birth, sex, migration (into, out of, and around the study area), and date of death (Figure 1). Each green spot represents an address and a table of the mentioned attributes.

The CPR contains data on more than 7 million people who are or have been residents in Denmark since 1968. The key to the register is the personal identification number, the CPR number, which is a unique 10-digit number that all residents in Denmark are assigned at birth or when immigrating. In addition to the present address of all residents, the CPR also contains historical addresses and the dates on which the individual moved to and from that address. If a person dies, disappears, or takes up residence abroad, this is also recorded as having moved away from the address. The CPR, its structure, updating, and other details are described elsewhere (Briggs et al. 2002).

All health registers in Denmark use the CPR number as entry key, which makes it easy to merge health data into a GIS where CPR has been incorporated. In this study all malignant cancer (except skin cancer) was used as the health outcome indicator. By merging the Danish Cancer Register with CPR data, the necessary cancer incidence information was retrieved. Details on Danish health registers have been described elsewhere (Briggs et al. 2002), particularly in the Danish Cancer Register (Storm 1991).

In this study, 1986–1998 was the time span chosen for analyses. The first year, 1986, was chosen as a compromise between the arduous work of geocoding historical addresses and the cost of this operation versus having a sufficient number of years with cancer data for analyses. The main dioxin producer, the aluminum recycling plant, became operational in 1970, which was sufficient time from start of production (and pollution) to account for the induction and latency time of developing (eventual) cancer in the surrounding population. The end of study year, 1998, was chosen because that was the last year with obtainable cancer data at the time a request was sent to the Danish Cancer Register.

In this study the exposure simulation model, OML, was used to demarcate three zones relevant for studying cancer development related to the dioxin exposure:

- Zone 1 encompassed the whole residential area identified to be exposed to dioxin.
- Zone 2 included the area identified to be exposed to 3.5 ng dioxin/m^3^/hr or higher. Zone 2 is part of zone 1.
- Zone 3 included the inner and highest exposed area with estimated dioxin immissions of 4.5 ng dioxin/m^3^/hr or higher. Zone 3 is part of zones 2 and 3.

**Figure 2:**
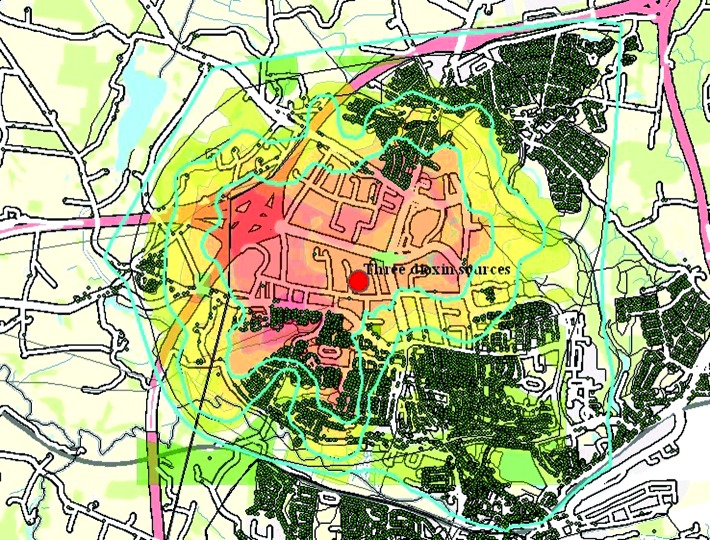
Computer-simulated exposure of dioxin from three sources (red) are layered onto the electronic map (GIS) and seen as different colored bands, with highest dioxin immissions in bright red and lowest in faint green. The immission concentration band borders (blue) are used to demarcate the three zones used for analyses of cancer development.

**Figure 3:**
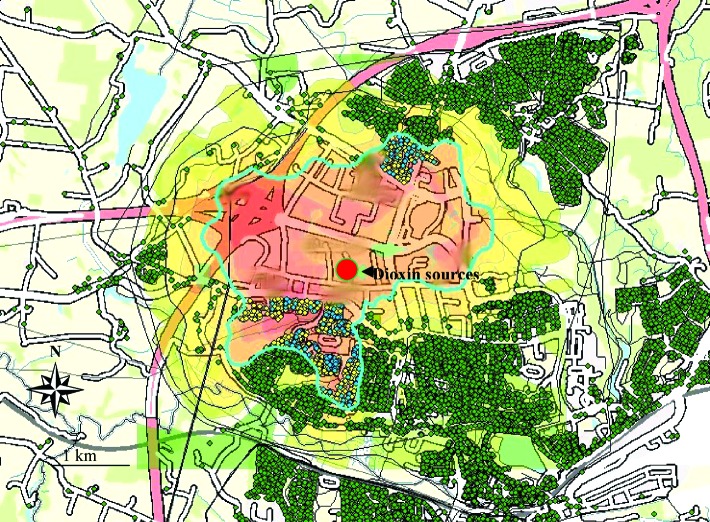
Demarcation of zone 3 and the addresses (and individuals) in blue within the polygon. Cancers diagnosed among the individuals in zone 3 during 1986–1998 are marked in yellow (overlayed on the blue dots).

These zones and the selection are illustrated in Figure 2, where the immission concentration band borders are used to demarcate the three zones. In Figure 3 the malignant cancers have been linked and overlayered, appearing as yellow dots. For confidentiality reasons, any single, outstanding cancer case has been obscured so that only clusters of cancers (aggregated over 13 years) are visible.

The following criteria were used to select the study population:

- Individuals were included if they lived in the area between 1986 and 1998. Individuals were included in a calendar year if they moved into the area on or after 1 January in the same year.
- Cancer cases were included if the year of diagnosis was in or later than the year the individual moved into the area (i.e., cancer cases with year of diagnosis before migration into the area were excluded).
- Skin cancer diagnoses (ICD-7 code 191; National Board of Health 2003) were excluded because they comprise a considerable number of rather harmless cancers.

The reference population chosen was the Danish population in the same period, 1986–1998. It was desirable but not financially possible to obtain a reference area similar to the study area.

### Study Population

At the start of follow-up, 1 January 1986, 15,404 individuals resided in the study area. During the next 13 years, between 2,069 and 3,470 individuals moved into the area each year; a total of 46,392 different individuals resided in the area during the 13-year follow-up period. During the study period the population gradually increased to 20,217 individuals by the end of 1998. Among the 46,392 persons who lived in the study area from 1986 to 1998, 3,205 individuals were newborns who had their first-ever address in the area.

**Figure 4:**
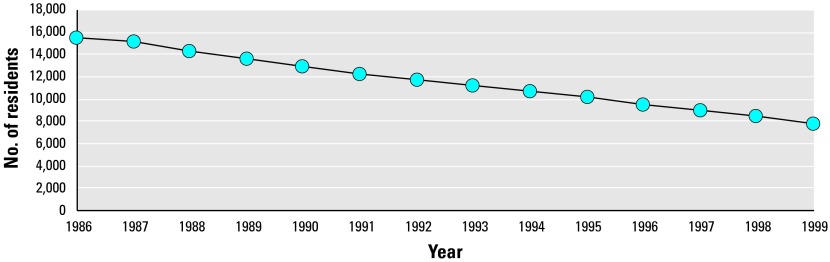
Migration of residents living in the study area in January 1986 from the area from 1986 to the end of 1998.

Among the 15,404 individuals residing in the study area 1 January 1986, 7,758 (50.4%) were still living in the study area at the end of 1998, and 4,799 (31.2%) had not changed their address. Figure 4 illustrates this development. Among those who were < 10 years of age on 1 January 1986, 57% were still residing in the area 13 years later, whereas only 30% of those 10–20 years of age still lived in the area at the end of 1998. This figure was 43% for the group 21–30 years of age; 65% for the group 31–40 years of age, and increased to 71% for the group 41–50 years of age. The same figure gradually decreased for the group 51–60 years of age to 65% for those who remained in the area until the end of the study period, and rapidly decreased for older age groups.

The 46,392 individuals who lived in the study area had a total of 75,437 periods of residence. Among the same 46,392 individuals, 61.5% had one address, 21.5% had two different addresses, and 17% had three or more addresses within the study area during 1986–1998.

On 1 January 1986, 50.3% of the females and 55.8% of the male residents in the study area were < 40 years of age. The corresponding figures for the whole population of Denmark in 1986 were 54.0% for male residents and 58.3% for females.

### Statistical Methods

For each year from 1986 through 1998, information on the number of eligible residents and cancers from the study area (zones 1–3) were retrieved through the GIS model and cumulated into nine 10-year age bands (0–9, 10–19, 20–29 . . . ≥80) stratified by sex.

The Danish population during the same period was used as the reference population. Number of cancers was derived from the Danish Cancer Register and population data were from the Bureau of Statistics (Danmarks Statistik). These data were similarly grouped into cumulated 10-year age bands stratified by sex. Years of risk were calculated using the number of residents in each calendar year in the study area (“living in and not moved out”) and in the general population, respectively. Each resident in a given year was counted as 1 year of exposure. The number of expected cases of cancer was calculated based on the total number of person-years for each 10-year age category multiplied by the cancer rate of the Danish men and women, respectively, during the same period. The standardized incidence ratio (SIR)—the ratio between observed and expected numbers—was calculated with 95% confidence limits (95% CL) assuming a Poisson distribution of the cancer cases. Normal distribution for observed cancers was assumed when figures were above 100.

## Results

The method of using an air pollution simulation model to identify exposure and exposed population was operational, and the subsequent incorporation into a GIS environment integrating individual statistics of address, vital statistics, and cancer created no severe technical problems.

Results of the statistical analyses are presented in Table 1. Only a single age band in zone 1 had confidence limits above 1.0. No excess of cancer in the study area during 1986–1998 could be demonstrated. The study population was anticipated to be geographically stable, but this appeared not to be true, with only one-third of the original residents still living in the area at the end of the study period.

## Discussion

The OML, a commercial product (Danish Environmental Protection Agency 1997), is used widely by environmental regulatory bodies in Denmark to assess immission values of airborne pollutants. This product proved useful to visualize exposure in a GIS milieu to outline the research area. The incorporation of the model in GIS presented no serious technical problems.

However, the OML output, like with most models, is no better than the quality of the input data, and in this case only two dioxin measurements from the chimney smoke were available. In 2000, when the environmental authorities discovered grossly excessive emissions (180 ng dioxin/m^3^/hr) with a legal threshold of 1 ng dioxin/m^3^/hr, the aluminum recycling plant immediately started injecting active carbon and chalk into the smoke-cooling process, hence reducing the content of dioxin to far below thresholds. The emission of dioxin has been reduced further since then. The official threshold was lowered in 2001 to 0.1 ng dioxin/m^3^/hr, following EU regulations. So the first and only data available were two measurements in autumn 2000, which do not allow for extensive conclusions on the amount of airborne dioxin dispersed to the adjacent surroundings.

Airborne dioxin alone is adsorbed onto plants, trees, vegetables, and soil but is easily washed away by rain. A soil examination in the exposed area in the summer 2001 produced no evidence of a major contamination of the area (Vejle Amt 2001).

A major study on environmental and hereditably caused cancers (Lichtenstein et al. 2000) concluded that genetic factors make only a minor contribution to development of sporadic cancer, with environmental factors being the major contributor.

Airborne dioxin is presumably absorbed in the lungs, making up 75% of the total content. European average dioxin concentrations range between 0.01 and 0.4 pg/m^3^, which translated into a Danish situation for an average adult is an intake via the lungs of 0.2–8 pg dioxin/day. Dioxin via the airways is not the only entrance into the body; intake via food is assumed to constitute as much as 15 pg a day (2.44 pg/kg body weight (bw)/day; 70 kg) (Danish Environmental Protection Agency 1997).

If people in Kolding have had concentrations of airborne dioxin in their ambient environment for many years in the range of the measured values of 100–200 ng dioxin/m^3^/hr that produce inhalation concentrations in the range of 1–6 pg dioxin/m^3^/hr, then using the above estimate would have caused a daily extra intake of 20 pg dioxin in the least polluted area and up to 120 pg in the highest polluted area (zone 3).

Tolerable daily intake is 5 pg/kg bw in Denmark (Danish Environmental Protection Agency 1997). In the Netherlands authorities recommend figures be lowered to 1 pg/kg bw (Health Council of the Netherlands 1996). An extra intake of up to 120 pg dioxin/day for an adult would entail a substantial extra burden for the body of a well-known carcinogen.

In the Kolding case no one knows whether actual emissions over the years have been even higher (or lower) than the measured values, meaning that the measured values could just as well have been in the lower range of the actual pollution of dioxin. However, the information above on dioxin in soil in the exposure area (Vejle Amt 2001), together with the fact that no excess cancers were detected in any of the years under study, in any of the zones, in any age group or any sex group, indicate that no major pollution of the study area with airborne dioxin has taken place over the years. As the peak dioxin values were detected in late 2000, any later consequence on cancer development will not be detectable until later. The relevant authorities have decided to continuously scrutinize the cancer data of the area in years to come.

Four years of latency has been chosen as a very conservative restriction to allow for any early effect. Most likely the latency, at least for adults, is much longer.

A planned follow-up of the present study is a search in the Danish Cancer Register for cancers diagnosed outside the study area among previous residents of the study area.

The tool we have developed has its limitations. Most environmental exposures in a modern industrial society stem from food or are widely present in the environment, for example, exhaust from vehicles. Fewer are present in well-defined geographical areas, and few are strong enough to have any significant impact on health. These factors limit the opportunity to investigate environmental health relationships using spatial analytical methods, and inhibit the types of problems that can be addressed.

The Address Project offers new and unique possibilities for performing studies of relationships between environmental exposures and health of the population in Denmark. These studies might be based on a range of different study designs (Aylin et al. 1999; Elliott et al. 1992). Because of the ability to track individuals over time, retrospective, space–time studies are possible. In each case, the detailed address-based data now available and the ability to link data files are likely to enhance these studies.

In this study we decided to use the knowledge of the migration of the population to apply two restrictions: to include only individuals who had actually stayed in the area and to include only the cancer cases that were diagnosed after the individual had moved into (or after their birth in) the area.

Further restrictions could have been implemented, but this would have implicated the choice of a reference area with a population where the same restrictions could be made. The actual restrictions that were applied based on the individual migration data available are an improvement in dealing with this misclassification problem in epidemiologic studies and indicate the vast opportunities in the system.

There are, however, several important limitations on what can be expected even from a fully developed system, and several issues concerning choice of study design need to be carefully considered.

First, it is important to base such studies on defined and plausible hypotheses about the relationships being examined. Possible exposure pathways also need to be identified. Without these preconditions, results are likely to be difficult to interpret, at best, or are even misleading.

Second, by considering environment, one is concerned with more than just the soil on which we walk, the water we drink, and the air we inhale. Environment is also what we eat and wear, what we smoke, where we work and relax, and from a biological point of view, it is more likely that the causes of death and diseases may be found here rather than geographically varying pollution in the ambient environment.

In addition to these theoretical considerations, a number of other limitations must be recognized. For example, most geographically based studies assume that populations are static and that exposures occur in fixed locations (usually the place of residence). Obviously, this is not true. People are highly mobile, both in terms of short-term activities (e.g., daily travel to work) and long-term migration. Rates of migration in a population may be high. Therefore, knowledge about where people work or have worked is essential if all misclassification is to be ruled out.

The analysis of the stability of the population in the study area disclosed a high mobility. Less than one-third lived at the same address after 13 years of observation, and only half were still residents in the study area. A high degree of mobility within the study area was also found. The chosen study area is an ordinary mixed residential and industrial suburb, and the observed mobility of the population is likely to be representative of similar areas in Denmark. In ecologic studies, information on exposure and the exposed individuals is of vital importance. Such studies will therefore be highly susceptible to the fact that only a relatively small proportion of the study population remains in the area during a prolonged exposure in the local environment. A further improvement in exposure assessment would be to measure actual at-risk time for each individual. This was not done in this study although it is possible within the model and with the Danish data sets. We hope to perform such a study in the future.

In an extensive analysis of geographic exposure modeling and its usefulness in environmental epidemiology (Beyea and Hatch 1999), the authors emphasized the importance of considering all uncertainty aspects when making the models: type and quantity of pollutants, their pathways into surroundings, exposed population, and time of pollution.

The tested GIS with linkage of addresses and individual health information gives new opportunities for high-quality, small-area health studies in a wide range of situations. When fully developed and covering the whole of Denmark, it will create a useful tool both for administrators, planners, and public health offices as well as researchers. As with all such systems, however, it is crucial to recognize the limitations of the system and to apply it only where appropriate. The geographical stability of the study population is especially crucial to address, describe, and include in the exposure assessment. Otherwise, this misclassification may totally distort the true picture.

## Figures and Tables

**Table 1:** Cumulated cancer incidence data from 1986 to 1998 for three zones.

Age group (years)	Men (*N*)*^a^*	Men (*n*)*^b^*	Expected (*n*)*^c^*	RR	95% CL	Women (*N*)*^a^*	Women (*n*)*^b^*	Expected (*n*)*^c^*	RR	95% CL
Zone 1: whole study area (estimated exposure > 0 pg dioxin/m^3^/hr)										
0–9	18.420	4	3.21	1.25	0.34, 3.19	16.871	3	2.57	1.17	0.24, 3.41
10–19	18.609	2	3.46	0.58	0.07, 2.09	18.990	7	2.64	2.65	1.07, 5.47
20–29	33.031	12	14.78	0.81	0.42, 1.42	33.448	11	13.98	0.79	0.39, 1.41
30–39	23.072	12	17.74	0.68	0.35, 1.18	21.994	18	27.14	0.66	0.39, 1.05
40–49	18.870	32	33.02	0.97	0.66, 1.37	19.357	59	66.47	0.89	0.68, 1.15
50–59	14.161	54	70.45	0.77	0.58, 1.00	15.113	106	97.75	1.08	0.71, 1.55
60–69	10.395	143	132.29	1.08	0.91, 1.27	12.930	138	148.67	0.93	0.64, 1.27
70–79	7.647	156	179.30	0.87	0.74, 1.02	11.560	176	171.68	1.03	0.74, 1.36
80+	3.421	65	95.76	0.68	0.52, 0.87	7.178	103	115.11	0.89	0.58, 1.28
Total	147.626	480	550.01	0.87	0.80, 0.95	157.441	621	646.01	0.82	0.82, 1.12
Zone 2 (estimated exposure > 3.5 pg dioxin/m^3^/hr)										
0–9	4.913	0	0.86	0.00	0.00, 4.31	4.485	2	0.68	2.92	0.35, 10.56
10–19	4.945	0	0.92	0.00	0.00, 4.01	4.723	2	0.66	3.05	0.37, 11.01
20–29	5.286	4	2.37	1.69	0.46, 4.33	5.387	3	2.25	1.33	0.27, 3.89
30–39	4.678	5	3.60	1.39	0.45, 3.24	5.335	2	6.58	0.30	0.04, 1.10
40–49	4.366	9	7.64	1.18	0.54, 2.24	5.053	14	17.35	0.81	0.44, 1.35
50–59	3.548	15	17.65	0.85	0.48, 1.40	3.792	29	24.53	1.18	0.79, 1.70
60–69	2.565	40	32.64	1.23	0.88, 1.67	3.141	35	36.12	0.97	0.68, 1.35
70–79	1.649	45	38.66	1.16	0.85, 1.56	2.596	35	38.55	0.91	0.63, 1.26
80+	600	13	16.79	0.77	0.41, 1.32	1.368	19	21.94	0.87	0.52, 1.35
Total	32.550	131	121.13	1.08	0.41, 1.32	35.850	141	148.66	0.95	0.66, 1.30
Zone 3 (estimated exposure > 4.5 pg dioxin/m^3^/hr)										
0–9	1.824	0	0.32	0.00	0.00, 11.62	1.492	0	0.23	0.00	0.00, 23.28
10–19	1.746	0	0.32	0.00	0.00, 11.36	1.543	1	0.21	4.66	0.12, 25.99
20–29	1.630	1	0.73	1.37	0.03, 7.64	1.740	0	0.73	0.00	0.00, 7.28
30–39	1.703	3	1.31	2.29	0.47, 6.70	1.993	1	2.46	0.41	0.01, 2.27
40–49	1.558	4	2.73	1.47	0.40, 3.76	1.943	6	6.67	0.90	0.33, 1.96
50–59	1.498	5	7.45	0.67	0.22, 1.57	1.667	12	10.78	1.11	0.58, 1.94
60–69	1.132	15	14.41	1.04	0.58, 1.72	1.586	16	18.24	0.88	0.50, 1.42
70–79	855	19	20.05	0.95	0.57, 1.48	1.295	12	19.23	0.62	0.32, 1.09
80+	292	4	8.17	0.49	0.13, 1.25	462	5	7.41	0.67	0.22, 1.57
Total	12.238	51	55.49	0.92	0.68, 1.21	13.721	53	65.96	0.80	0.60, 1.05

Abbreviations: 95% CL, 95% confidence limit; RR, relative risk.

**a**Background population.

**b**Number of cancer incidents.

**c**Expected numbers of cancer cases.
